# New perspective on acute boutonniere deformity: An anatomical study with illustrative case report

**DOI:** 10.1016/j.jham.2026.100490

**Published:** 2026-07-30

**Authors:** Lorenzo Andrea Ferrazzini, Thomas Giesen

**Affiliations:** Faculty of Biomedical Sciences, Università della Svizzera italiana, Via Buffi 13, Lugano, 6900, Switzerland

**Keywords:** Boutonniere deformity, Extensor tendon lesion, Proximal interphalangeal joint deformity, Acute boutonniere, Central slip

## Abstract

Acute boutonniere deformity is a heterogeneous clinical entity that has traditionally been attributed to rupture of the central slip of the extensor mechanism, with secondary palmar migration of the lateral bands. The temporal relationship between central slip detachment and lateral band displacement remains incompletely defined. We report a clinical case in which a patient presented with an immediate, actively irreducible boutonniere deformity caused by an uncommon injury pattern, characterized by longitudinal disruption of a single lateral band and rupture of the ipsilateral collateral ligament, with the central slip largely intact. The deformity could not be reduced by closed means and required surgical treatment.

Motivated by this observation, we performed an exploratory anatomical study on 16 cadaveric fingers. Isolated lesions of the central slip or of a single lateral band did not, in this model, produce an actively irreducible boutonniere deformity. By contrast, combined injuries consisting of a longitudinal lesion of one lateral band and rupture of the ipsilateral collateral ligament consistently resulted in immediate palmar displacement of the lateral band below the axis of the proximal interphalangeal (PIP) joint, irrespective of central slip integrity.

These exploratory findings suggest that, alongside the classical mechanism of central slip rupture, an under-recognized injury pattern combining longitudinal lateral band disruption and ipsilateral collateral ligament failure may produce an immediate, actively irreducible boutonniere deformity, with potential implications for early diagnosis and treatment. Further clinical validation is required.

**Level of evidence:**

IV

## 1 Introduction

Boutonniere deformity is a complex and disabling condition of the hand, characterized by flexion at the proximal interphalangeal (PIP) joint and hyperextension at the distal interphalangeal (DIP) joint, which is difficult to correct once it has fully developed. Classically, central slip detachment, with concomitant injury to the spiral fibers and the triangular ligament, has been considered the primary lesion underlying the development of boutonniere deformity, followed by progressive palmar displacement of both lateral bands. Traumatic detachment of the central slip is thought to destabilize the lateral bands, which gradually migrate palmarly and begin to act as PIP flexors and DIP extensors, producing the characteristic deformity.^1^, ^2^, ^3^

It remains incompletely defined whether palmar displacement of the lateral bands occurs acutely at the time of injury, subacutely (2–3 weeks later), or only as a late phenomenon. This issue is still debated and described in non-uniform terms, even in recent international textbooks. Most of the literature on acute and chronic boutonniere deformity that informs current teaching is largely derived from studies published in the 1960s, 1970s, and 1980s, with comparatively limited integration of more recent anatomical and biomechanical evidence.^4^

In current clinical practice, the term acute boutonniere deformity is often applied to a zone III extensor tendon injury presenting with a flexed PIP joint, typically without fixed hyperextension of the DIP joint. In this context, the Elson test^5^ is widely used: increased resistance to passive DIP flexion when the PIP joint is held in flexion is interpreted as proximal retraction of the central slip, rather than as irreducible palmar migration of the lateral bands.

This raises a clinically relevant question: does isolated rupture of the central slip alone acutely produce a true boutonniere deformity, or is the classical deformity in most cases a delayed manifestation?

A growing body of evidence suggests that isolated central slip injury may not be sufficient to produce a true acute boutonniere deformity. Cadaveric models 6, 7, 8 as clinical investigations 9, 10, 11, 12 have repeatedly shown that isolated central slip detachment rarely results in an immediate, actively irreducible boutonniere deformity. Boutonniere deformity therefore appears to result from a multifactorial interplay of anatomical structures and biomechanical mechanisms.

In parallel, several clinical and anatomical studies have described acute irreducible deformities of the PIP joint associated with collateral ligament disruption and abnormal displacement of the lateral band, often reported under the term irreducible volar rotatory subluxation of the PIP joint.^13^, ^14^, ^15^These injuries may present with fixed PIP flexion and extensor mechanism dysfunction, contributing to diagnostic and terminological ambiguity in the acute setting and raising the question of whether they represent a distinct joint pathology, a specific mechanism of acute boutonniere deformity, or overlapping entities.

In the present manuscript we report a clinical case of a closed injury to the little finger that resulted in an immediate, actively irreducible boutonniere deformity, in which intraoperative findings showed a longitudinal split of the extensor apparatus in zone III, palmar displacement of a single lateral band, and avulsion of the ipsilateral collateral ligament, while the central slip remained largely intact. Early surgical intervention permitted direct intraoperative observation of an acute irreducible boutonniere deformity not driven by central slip detachment but rather by traumatic palmar displacement of a single lateral band associated with lateral joint instability.

Motivated by this clinical observation, we undertook an exploratory anatomical study on 16 cadaveric fingers to investigate the biomechanical contribution of individual components of the extensor mechanism to the development of an acute, actively irreducible boutonniere deformity. Using cadaveric specimens, we simulated isolated and combined injuries of the central slip, lateral bands, and collateral ligaments. The aim was to identify which anatomical lesion, or combination of lesions, was associated with immediate, actively irreducible palmar displacement of the lateral band, and to better delineate the relationship between this pattern and other acute PIP joint injury patterns. Given the small sample size and the in vitro nature of the model, the study was designed and is presented as hypothesis-generating.

## 2 Materials and methods

We performed an exploratory cadaveric anatomical study using 16 fingers from four fresh frozen cadaveric hands, sectioned at the distal third of the forearm, and focusing on the extensor mechanisms of the four long fingers (index, middle, ring, and small fingers) of each hand. To ensure donor identity protection, all specimens were anonymized before analysis.

Only hands with intact fingers, free from visible pathologies or damage to the extensor apparatus, were selected to meet the anatomical requirements of the study. Ethical approval for the study protocol was obtained from the relevant ethics committee, and informed consent was secured from each donor.

The dissection process involved removal of the skin and subcutaneous tissue to fully expose the extensor mechanism of each finger. Once exposed, key anatomical structures, including the central slip, lateral bands, triangular ligament, transverse retinacular ligament, oblique retinacular ligament, and the collateral ligaments of the PIP joint, were carefully identified and documented.

To simulate pathological injuries and evaluate their biomechanical effects, two groups of fingers were studied using specific sequences of tendon injuries and measurements. During dissection and measurement procedures, the wrist was positioned in 20° of extension, and the metacarpophalangeal (MCP) joints in 20° of flexion. This positioning was chosen to minimize tension on the extrinsic extensor tendons while simultaneously avoiding tensioning of the intrinsic tendons.

To improve consistency, in the present manuscript we use the following operational terminology. Acute boutonniere deformity refers to the clinical phenotype of a recently injured finger presenting with a flexed PIP joint and impaired active extension, and extended DIP joint with limited active and passive flexion, without necessarily implying a specific lesion.

True acute irreducible boutonniere deformity refers to a finger in which the lateral band is displaced palmar to the PIP joint axis and (i) cannot be repositioned dorsally above the joint axis by simulated active extensor and intrinsic activation in our cadaveric model (or by active extension by the patient in vivo) and (ii) is not repositioned dorsally above the axis even when the joint is passively extended, so that on subsequent flexion the lateral band returns palmar to the axis. Importantly, passive extension of the joint may still be anatomically feasible. The term irreducible therefore refers to the position of the lateral band relative to the PIP joint axis of rotation, not to the static range of motion of the joint itself. We underline that this definition is experimental and specific to the present model and is not proposed as a universally accepted clinical definition.

Volar rotatory subluxation of the PIP joint refers to the radiographic and biomechanical configuration described by Journé et al.^14^ and Tohyama and Konishi,^15^ which can produce a clinical phenotype overlapping with acute boutonniere deformity. The term pseudo-Stener-like effect is used descriptively to indicate displaced lateral band entrapment beyond the joint axis between the collateral ligament and the joint.

### 2.1 Group 1: Central slip, lateral band, and collateral ligament injuries

**Fig. 1:**
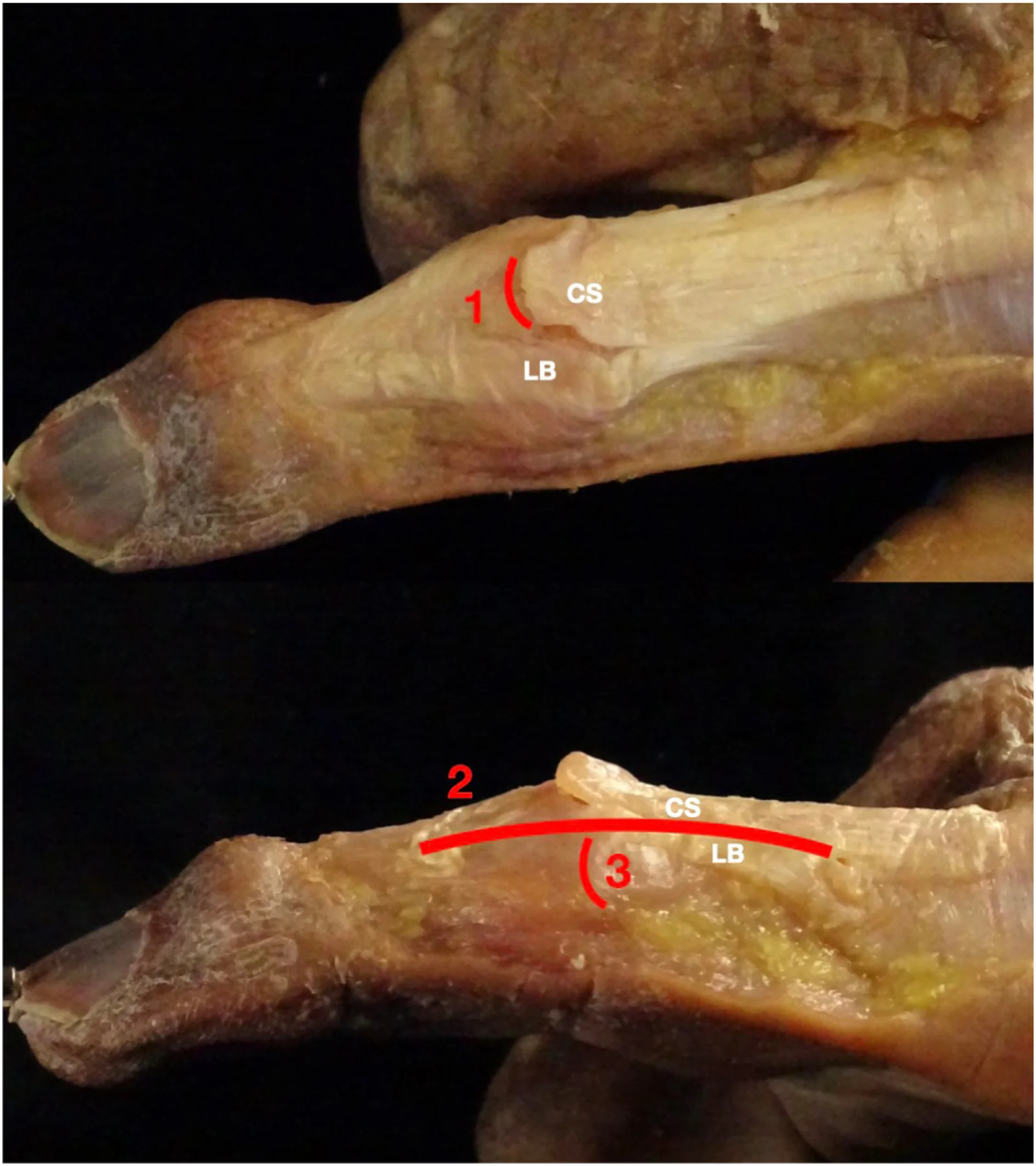
Group 1 lesions patterns: 1) Central slip detachment, 2) Longitudinal lesion of a lateral band, 3) Lesion of a collateral ligament, CS: central slip, LB: lateral band.

In Group 1, eight fingers (two each of the index, middle, ring, and small fingers) were subjected to the following sequence of lesions:

A transverse division of the central slip was performed at the level of the dorsal expansion and partially excised, creating a 5 mm defect to simulate complete central slip detachment. Subsequently, a longitudinal separation of one lateral band from the central slip was carried out on either the ulnar or radial side of the same finger. This longitudinal separation extended proximally for 15 mm from the PIP joint, affecting approximately 50% of the oblique fibers of the intrinsic hood. Distally, the triangular ligament was likewise compromised by 50%, though not completely disrupted. Finally, a lesion of the collateral ligament on the same side as the lateral band separation was created (Fig. 1).

### 2.2 Group 2: Lateral band and collateral ligament injuries

**Fig. 2:**
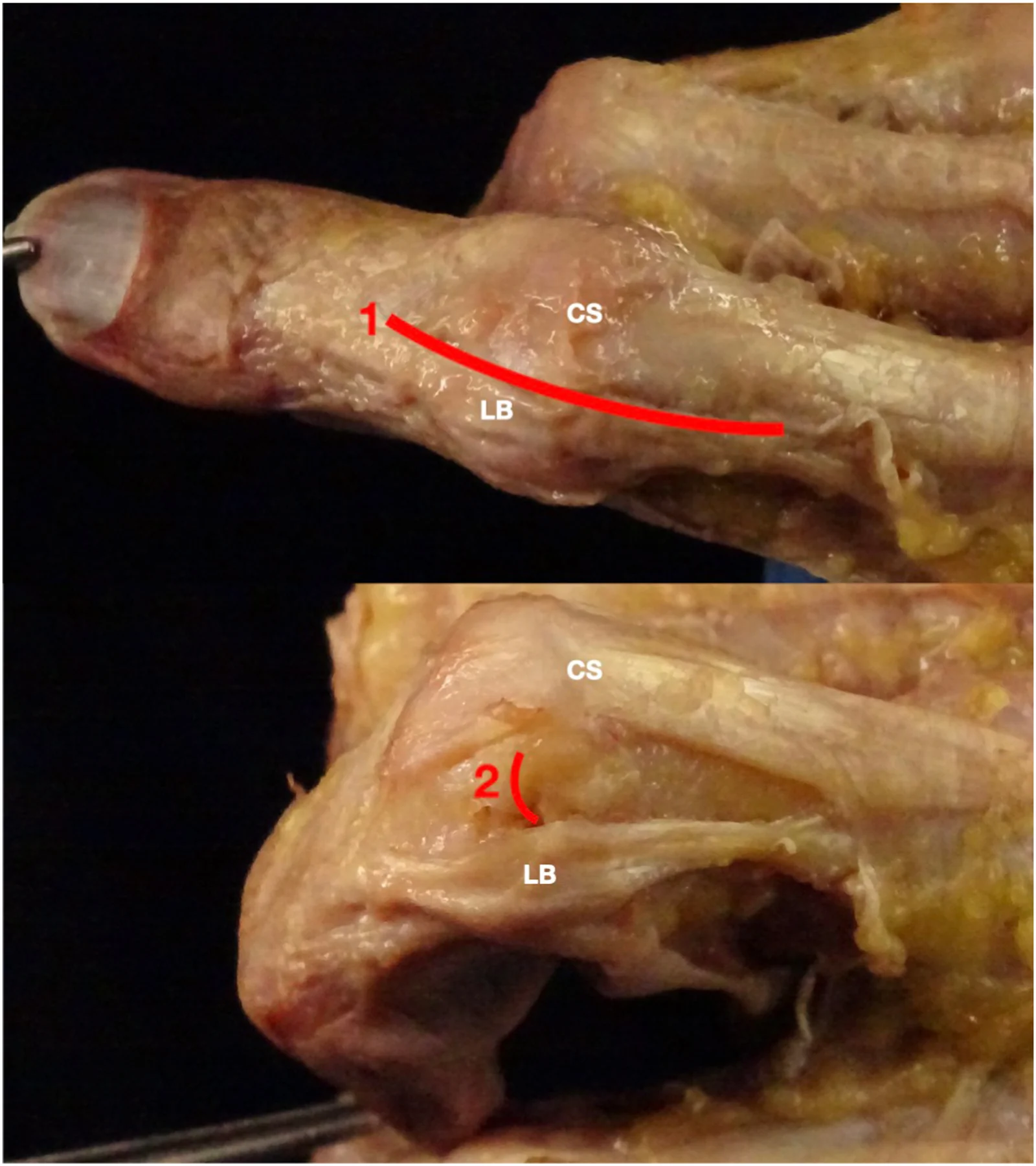
Group 2 lesions patterns: 1) Longitudinal lesion of a lateral band, 2) Lesion of a collateral ligament, CS: central slip, LB: lateral band.

In Group 2, another set of eight fingers (two each of the index, middle, ring, and small fingers) was subjected to the following sequence of lesions:

A longitudinal separation of one of the two lateral bands from the central slip was performed on either the ulnar or radial side of the finger across the PIP joint. This longitudinal separation extended proximally for 15 mm from the PIP joint, damaging approximately 50% of the oblique fibers of the intrinsic hood. Distally, the triangular ligament was similarly compromised by 50%, without being completely disrupted. Subsequently, a lesion of the collateral ligament on the same side as the lateral band separation was created (Fig. 2).

### 2.3 Observation and measurement of lateral band displacement

In both groups, the flexor, extensor, and intrinsic components were isolated in the palm and at the base of each finger. Each finger was passively flexed, and active flexion was then simulated by applying a constant force of 20 N to the FDP tendon. After each flexion, the finger was extended either passively or by applying a 20 N force to the relevant extensor tendon, and subsequently to one or both intrinsic tendon groups. Each simulation was performed twice, and lateral band measurements were recorded during the second repetition. Lateral band displacement was first observed in four randomly selected, uninjured fingers and was set as the reference physiological palmar displacement. For the purposes of the present study, a boutonniere deformity was operationally defined as “actively irreducible” when both of the following conditions were met after the lesion sequence: (i) simulated active activation of the extensor tendon, of the intrinsic tendons, or of both, failed to reposition the displaced lateral band dorsally above the center of rotation of the PIP joint (i.e., reduction was not achievable by simulated active forces) and (ii) passive extension of the joint, although still anatomically feasible, did not bring the displaced lateral band back above the axis of rotation, so that on subsequent passive flexion the lateral band returned palmar to the joint axis. We emphasize that this definition is experimental and specific to the present cadaveric model, was developed solely to standardize observations within the model, and is not proposed as a universally accepted clinical definition. Additionally, in both groups the position of the lateral bands was quantitatively measured after the first and the final lesion, with the flexor tendon under simulated active pull.

### 2.4 Data collection and analysis

Dynamic tendon displacements during flexion and extension were documented using a high-resolution digital camera (Nikon D850, Nikon Corporation, Tokyo, Japan; 2017). Recordings were performed from a lateral view of the finger, against a high-contrast background under enhanced illumination conditions to maximize visual clarity.

**Fig. 3:**
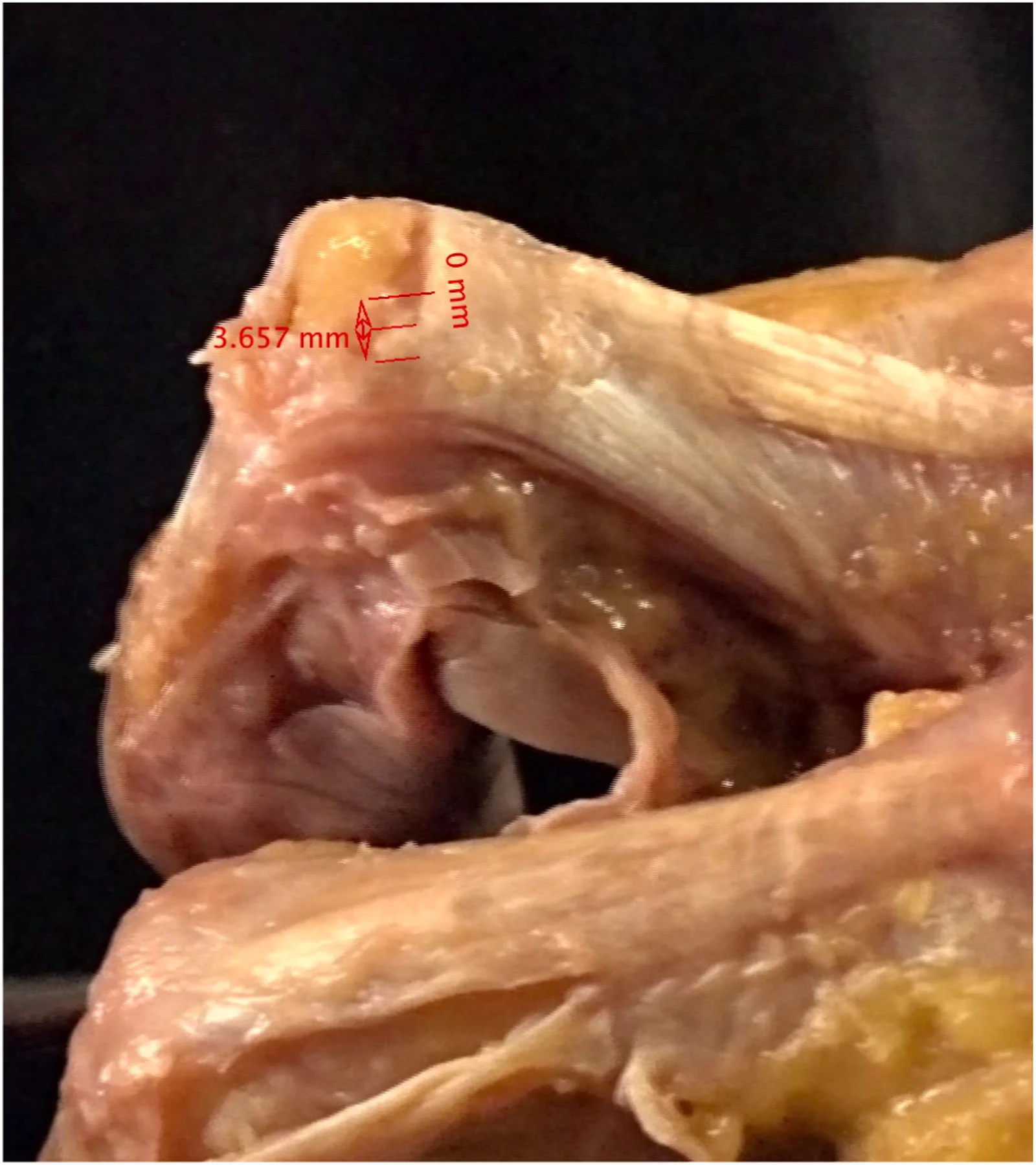
Tracker video analysis and modeling tool: measurement of tendon displacement.

Quantification of lateral band displacement was conducted using the Tracker Video Analysis and Modeling Tool (Version 6.3.0, D. Brown, R. Hanson, and W. Christian; 2025). Displacements were measured in millimeters and approximated to the nearest 0.5 mm. Measurements focused on the extent of palmar migration of the lateral bands, assessed relative to their original anatomical position in full finger extension (Fig. 3).

### 2.5 Statistical analysis

Given the small sample size and the exploratory nature of the study, no inferential statistical testing was performed. Lateral band displacement values are reported as descriptive statistics only (mean, standard deviation, and range), with the physiological palmar displacement of the lateral bands in the intact extensor system used as the reference value.

## 3 Results

In four randomly selected fingers, the physiological palmar displacement of the lateral bands was measured at 5 mm.

### 3.1 Group 1

- **Central Slip Detachment:**

**Fig. 4:**
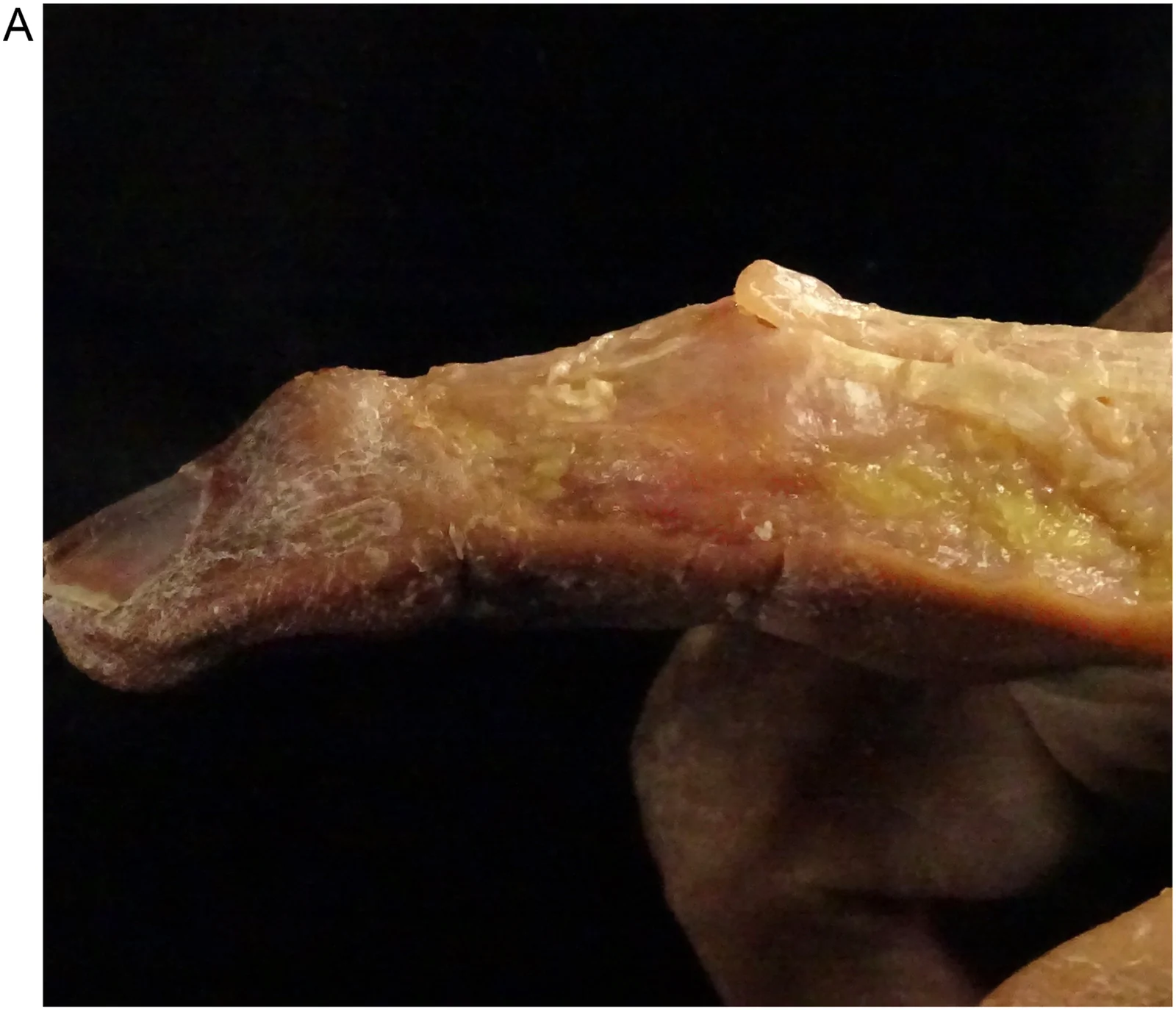

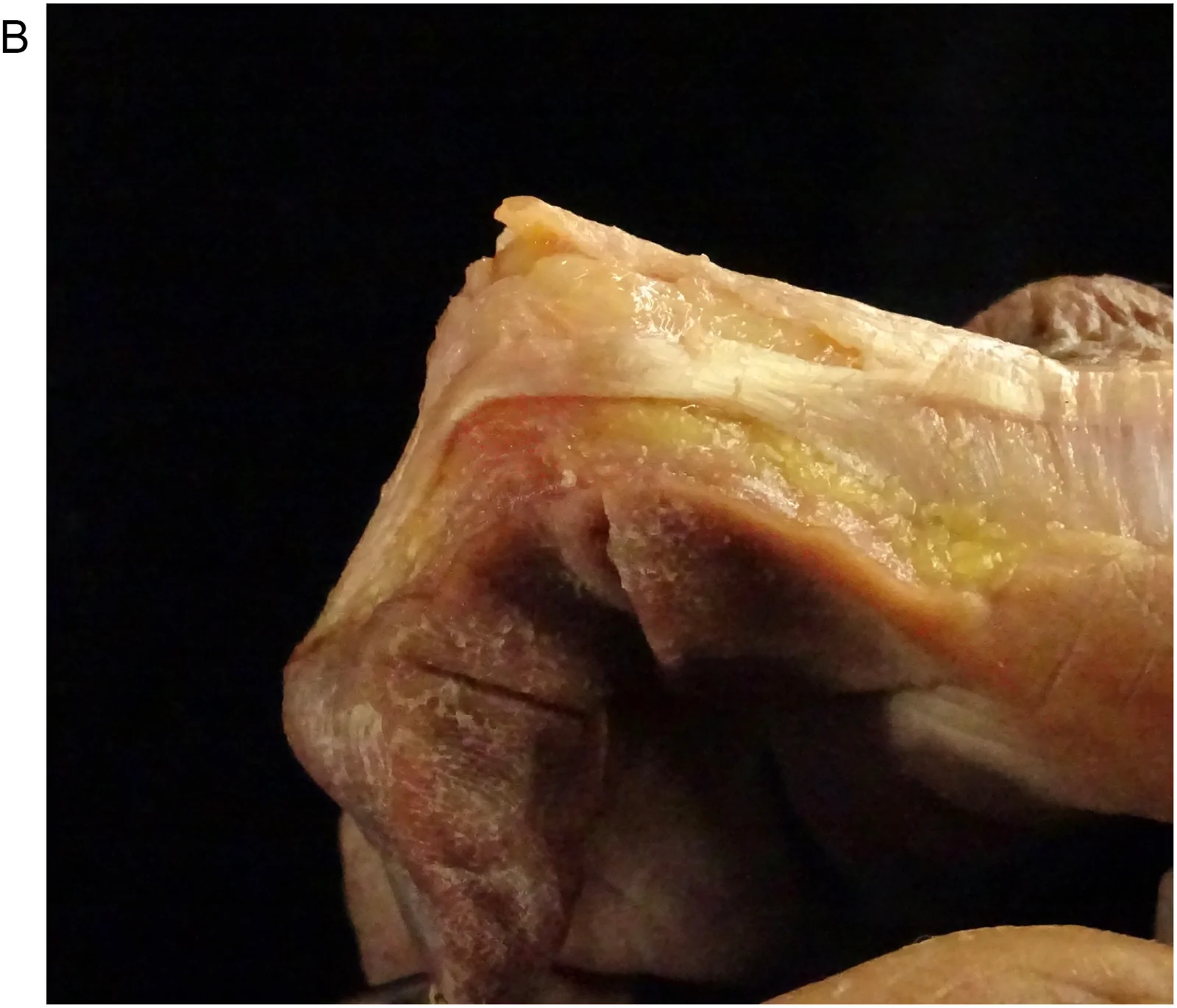
Finger sample from Group 1: a) central slip detachment with finger in extension, b) Negligible volar migration of the lateral band during PIP joint flexion.

Following the transverse injury of the central slip, the palmar displacement of the lateral band, measured with the MCP joint in 20° of flexion and the PIP joint in full flexion, averaged 4.75 mm (SD: 0.83; range: 3.5 mm - 6 mm) (Table 1). Isolated transverse central slip detachment never resulted in an irreducible boutonniere deformity, and no hyperextension of the DIP joint was observed. The lateral band consistently returned to its baseline alignment during both passive extension and simulated active extension, defined as a 20 N proximally applied force to both the intrinsic and extrinsic extensor components (Fig. 4 and Online Supplemental Video S1).

**Table 1:** Results: observations and measurements of Group 1, CS: central slip, LB: lateral band, CL: collateral ligament.

Finger	Lesion 1	Non reducible BD	LB volar movement (mm)	Lesion 2	Non reducible BD	Lesion 3	Non reducible BD	LB volar movement (mm)
III Left Specimen 1	CS	No	5.5	CS + LB	No	CS + LB + CL	Yes	10
V Left Specimen 1	CS	No	5	CS + LB	No	CS + LB + CL	Yes	11
II Right Specimen 2	CS	No	4.5	CS + LB	No	CS + LB + CL	Yes	13.5
IV Right Specimen 2	CS	No	3.5	CS + LB	No	CS + LB + CL	Yes	11
III Left Specimen 3	CS	No	5.5	CS + LB	No	CS + LB + CL	Yes	10
V Left Specimen 3	CS	No	4	CS + LB	No	CS + LB + CL	Yes	10
II Right Specimen 4	CS	No	4	CS + LB	No	CS + LB + CL	Yes	13.5
IV Right Specimen 4	CS	No	6	CS + LB	No	CS + LB + CL	Yes	13

Supplementary video related to this article can be found at doi:10.1016/j.jham.2026.100490

The following are the supplementary data related to this article:

Multimedia component 1 S1: Central slip detachment and marginal palmar migration of the lateral band.

**Central Slip Detachment + Longitudinal Lesion of a Lateral Band:**

**Fig. 5:**
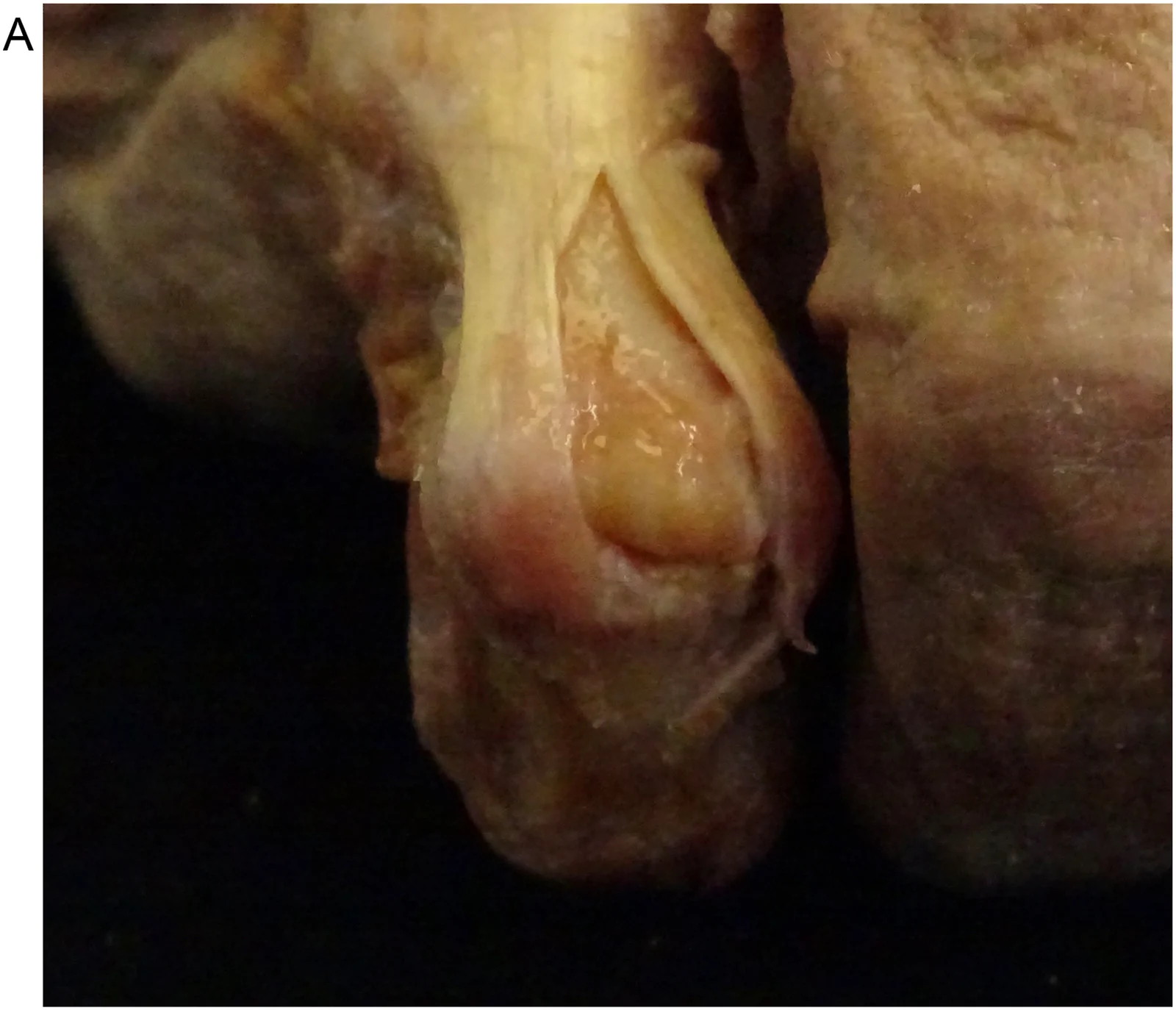

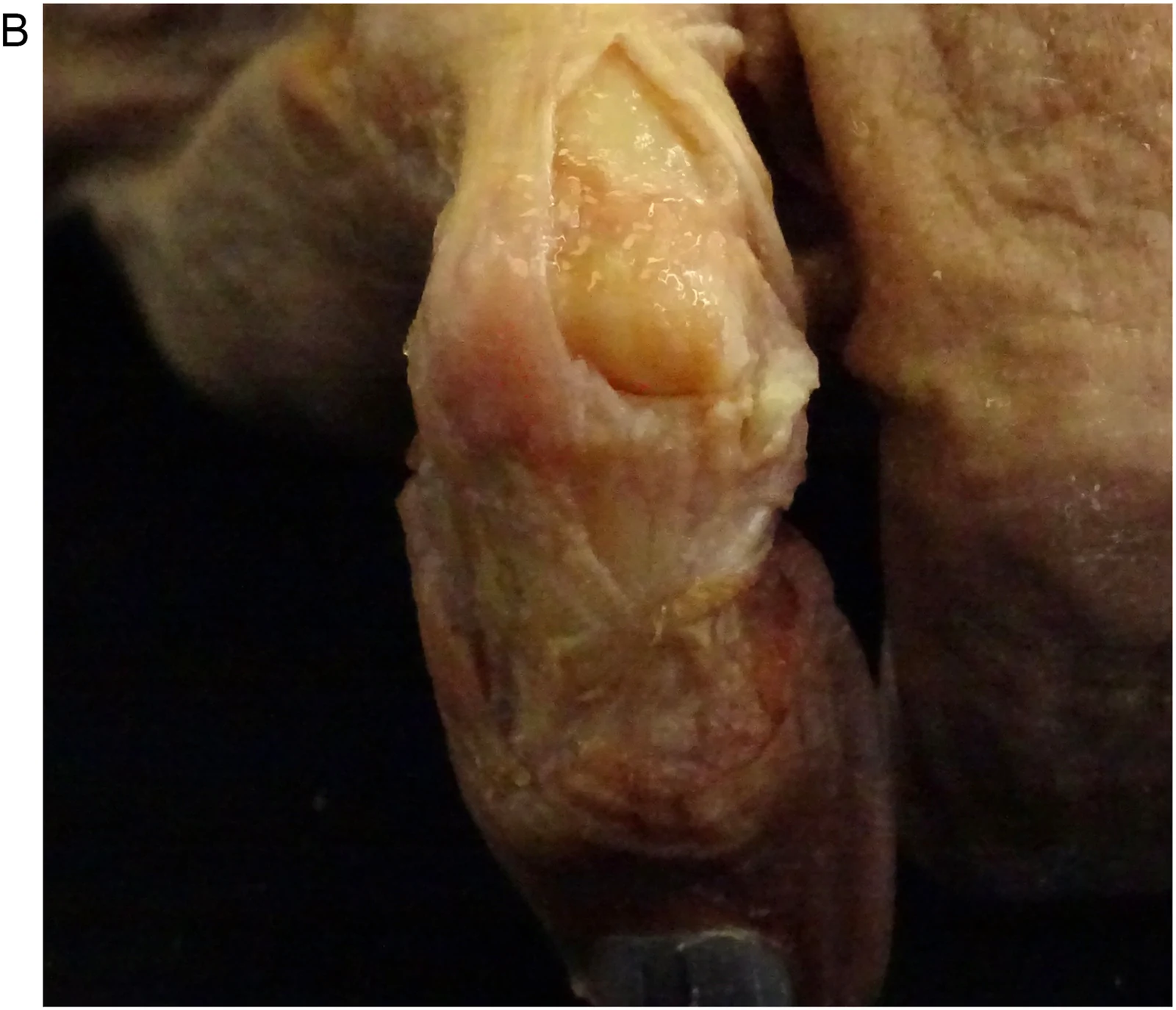

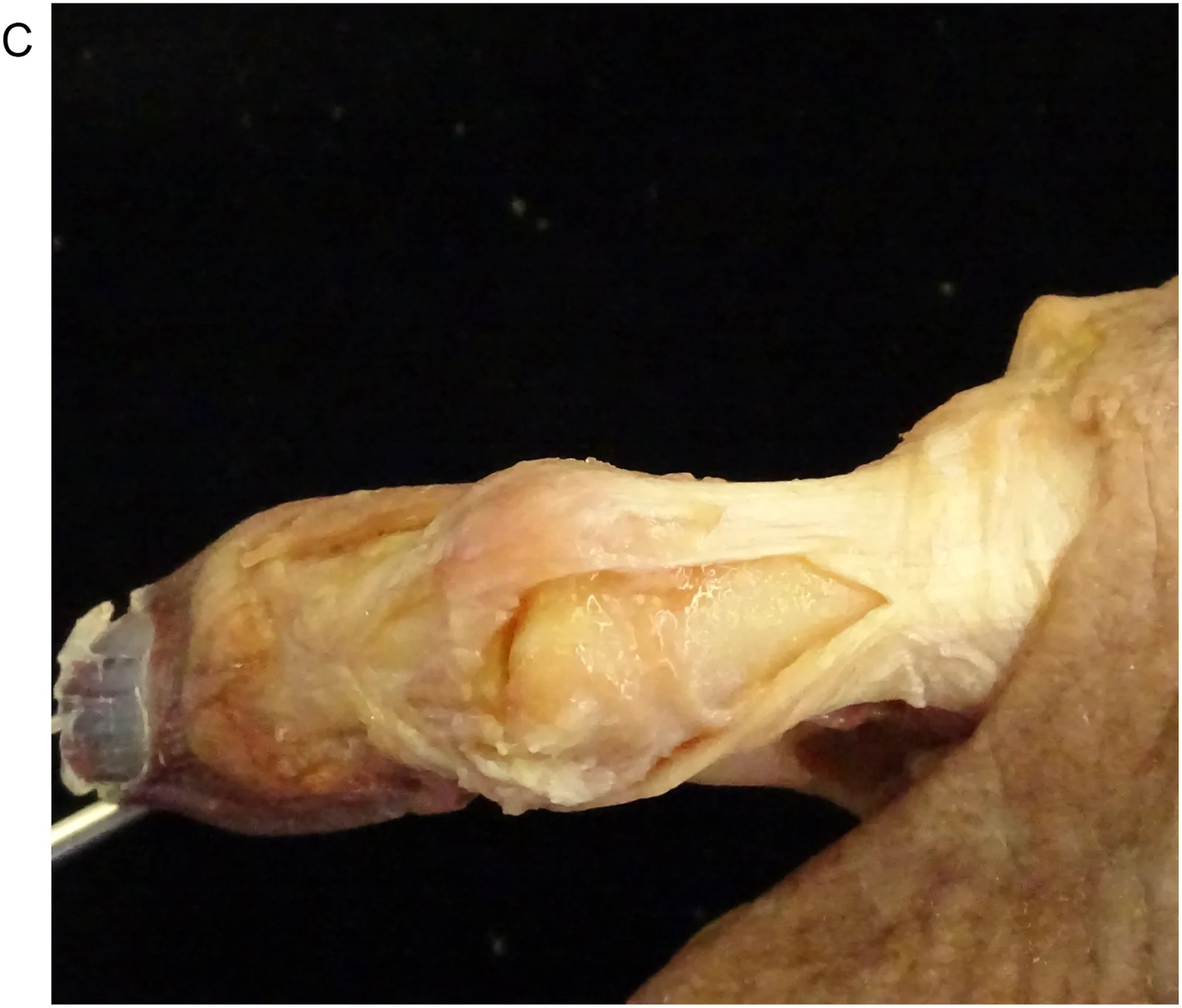
Finger sample from Group 2: a) Partial palmar migration of the lateral band, b) and c) Complete migration of the lateral band after collateral ligament lesion.

The addition of a longitudinal lesion of one lateral band to the existing transverse injury of the central slip did not result in an irreducible boutonniere deformity in any of the eight fingers tested (Fig. 5). No hyperextension of the DIP joint was observed.

- **Central Slip Detachment + Longitudinal Lesion of a Lateral Band + Lesion of a Collateral Ligament:**

When an ipsilateral collateral ligament lesion was added to the previous injuries, all fingers in this group developed an actively irreducible boutonniere deformity with extension of the DIP joint. The lateral band consistently exhibited pronounced volar displacement, migrating below the centre of rotation of the PIP joint. The mean displacement was 11.5 mm (SD 1.48; range 10.0–13.5 mm), substantially greater than the displacements observed in the isolated injuries described above. Neither passive extension nor simulated active extension could reduce the deformity. Notably, although passive extension of the PIP joint remained anatomically feasible, it did not return the lateral band dorsally above the joint axis. On subsequent passive flexion the lateral band consistently came back palmar to the axis. Reduction was achievable only when isolated traction was applied to the intrinsic tendon on the contralateral side of the lesion. Under simulated physiological conditions (combined traction of intrinsic and extrinsic tendons) the volarly displaced lateral band remained actively irreducible (Online Supplemental Video S2).

Supplementary video related to this article can be found at doi:10.1016/j.jham.2026.100490

The following are the supplementary data related to this article:

S2: Abrupt palmar migration of the lateral band after longitudinal lesion of it and concomitant lateral band disruption.Multimedia component 2

### 3.2 Group 2

- **Longitudinal Lesion of a Lateral Band:**

In these eight fingers, a longitudinal lesion between the central slip and one lateral band across the PIP joint did not produce an actively irreducible boutonniere deformity in any specimen. The separated lateral band exhibited a slight increase in volar displacement during passive and simulated active flexion compared with the isolated transverse central slip injuries of Group 1, with a mean displacement of 5.31 mm (SD 0.90; range 4.5–7.0 mm) (Table 2). As in Group 1, the injured lateral band consistently returned to its baseline position during both passive and simulated active extension (Fig. 5a; Online Supplemental Video S3). The difference in volar displacement between transverse central slip injuries (Group 1) and longitudinal injuries of a single lateral band (Group 2) was descriptively small and overlapped considerably between groups.

**Table 2:** Results: observations and measurements of Group 2, LB: lateral band, CL: collateral ligament.

Finger	Lesion 1	Non reducible BD	LB volar movement (mm)	Lesion 2	Non reducible BD	LB volar movement (mm)
II Left Specimen 1	LB	No	5.5	LB + CL	Yes	12.5
IV Left Specimen 1	LB	No	5	LB + CL	Yes	11.5
III Right Specimen 2	LB	No	5	LB + CL	Yes	11.5
V Right Specimen 2	LB	No	6.5	LB + CL	Yes	12.5
II Left Specimen 3	LB	No	4.5	LB + CL	Yes	9.5
IV Left Specimen 3	LB	No	4.5	LB + CL	Yes	10.5
III Right Specimen 4	LB	No	4.5	LB + CL	Yes	10.5
V Right Specimen 4	LB	No	7	LB + CL	Yes	12.5

Supplementary video related to this article can be found at doi:10.1016/j.jham.2026.100490

The following are the supplementary data related to this article:

S3: Longitudinal lesion of a lateral band without collateral injury: the lateral band migrates palmarly but can be reduced after activation of the intrinsic tendon or the intact central slip.Multimedia component 3

- **Longitudinal Lesion of a Lateral Band + Lesion of a Collateral Ligament:**

When a collateral ligament injury was added on the same side as the longitudinal separation of one lateral band from the central slip, all eight fingers developed an actively irreducible boutonniere deformity, characterized by palmar displacement of the injured lateral band below the center of rotation of the PIP joint, together with extension of the DIP joint. The mean lateral band displacement was 11.38 mm (SD 1.13; range 9.57–12.54 mm). Attempts to reduce the deformity through passive or simulated active extension were unsuccessful. Notably, although passive extension of the PIP joint remained anatomically feasible, it did not return the lateral band dorsally above the joint axis. On subsequent passive flexion the lateral band consistently came back palmar to the axis. Reduction was achievable only by selectively applying traction to the intrinsic tendon on the contralateral side of the injury. Under simulated physiological conditions with bilateral intrinsic traction, the volarly displaced lateral band remained actively irreducible (Fig. 5b and c; Online Supplemental Video S4). Compared with the isolated longitudinal lesion alone, the addition of collateral ligament injury produced a clearly larger and consistent palmar displacement of the lateral band across all specimens.

Supplementary video related to this article can be found at doi:10.1016/j.jham.2026.100490

The following are the supplementary data related to this article:

S4: Three-dimensional view of Group 2 finger specimen after palmar migration of the lateral band at the end of the experiment.Multimedia component 4

### 3.3 Other results

In group 1, during dissection performed under 4.3× magnification, it was very difficult, if not impossible, to separate the attachment of the central slip from the dorsal capsule of the PIP joint. In fact, we observed that the central slip becomes extremely thin just proximal to its insertion on the PIP joint, with a distinct change in consistency and color (Online Supplemental Video S5).

Supplementary video related to this article can be found at doi:10.1016/j.jham.2026.100490

The following are the supplementary data related to this article:

S5: Physiological mechanism of slight palmar dislocation of the lateral bands during passive PIP flexion. Note the marked thinning of the central slip, which acts as a connector for the lateral bands, and the formation of a very mild physiological boutonniere during flexion, with slight protrusion of the P2 head through the tendon structures.

In our specimens no palmar subluxation of the PIP joint was observed. No pseudo-Stener-like effect was observed.

## 4 Case report

A 34-year-old female patient presented with an acute boutonniere deformity and marked ulnar instability of the PIP joint of the left little finger. The symptoms developed following a traumatic twisting injury sustained six days prior to presentation. The patient had no relevant medical history.

On clinical examination, the PIP joint was held in a characteristic flexed posture consistent with boutonniere deformity and an inability to actively complete DIP flexion. Manual stress testing revealed significant ulnar instability of the PIP joint, quantified at approximately 35° under lateral stress, without visible ulnar deviation at rest.

**Fig. 6:**
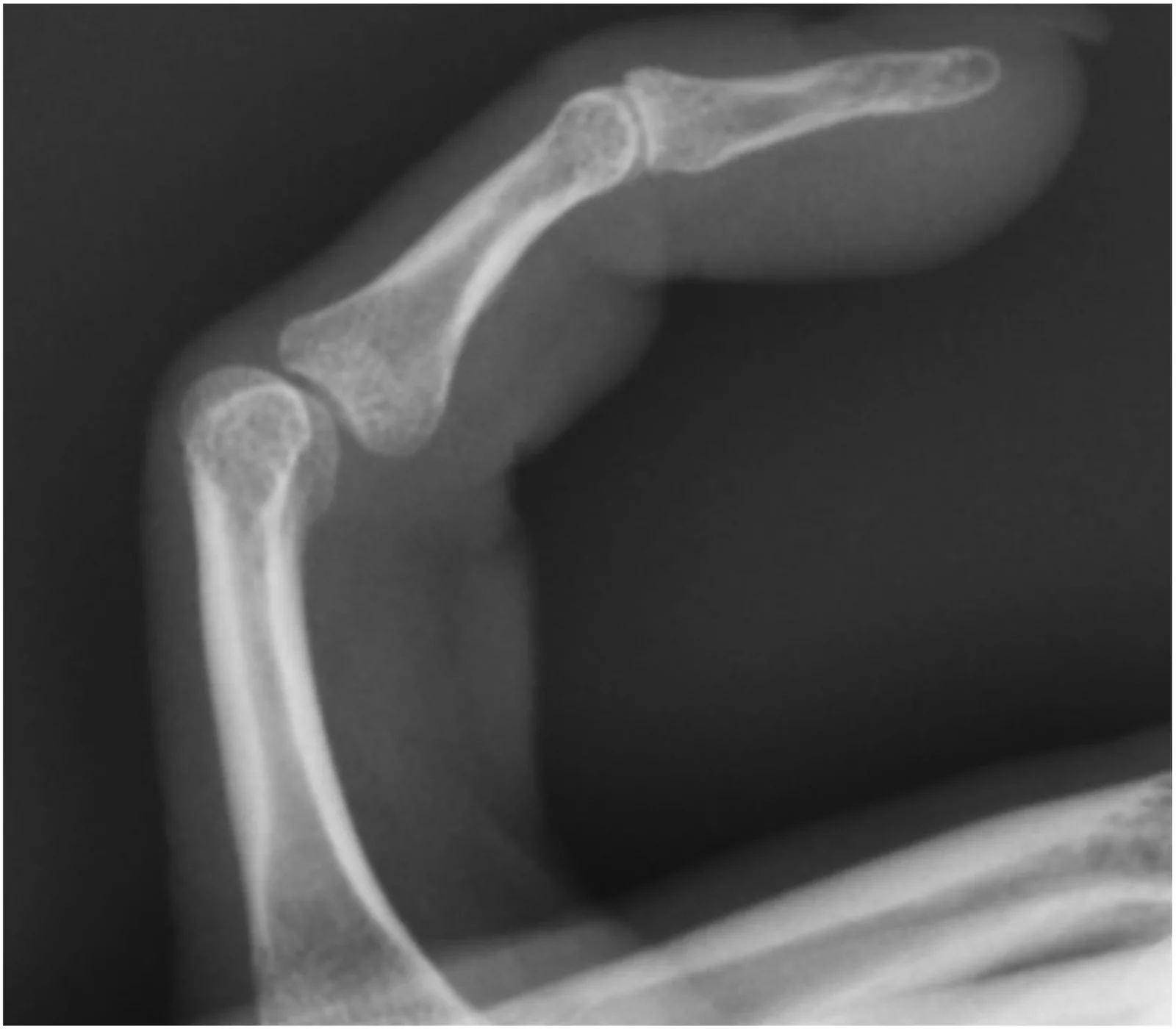
Lateral radiograph of the left little finger demonstrating PIP joint flexion with preserved joint congruency.

Standard lateral radiographs of the left little finger demonstrated a flexed PIP joint posture without evidence of fracture, joint subluxation, or incongruity of the articular surfaces (Fig. 6). An attempt at closed reduction was performed but failed to correct the deformity or restore joint stability.

Ultrasound imaging confirmed a rupture of the radial collateral ligament, with displacement of the torn ligamentous structures toward the joint line, reflecting the extent of ligamentous disruption responsible for the observed instability. Based on these findings, surgical intervention was indicated.

**Fig. 7:**
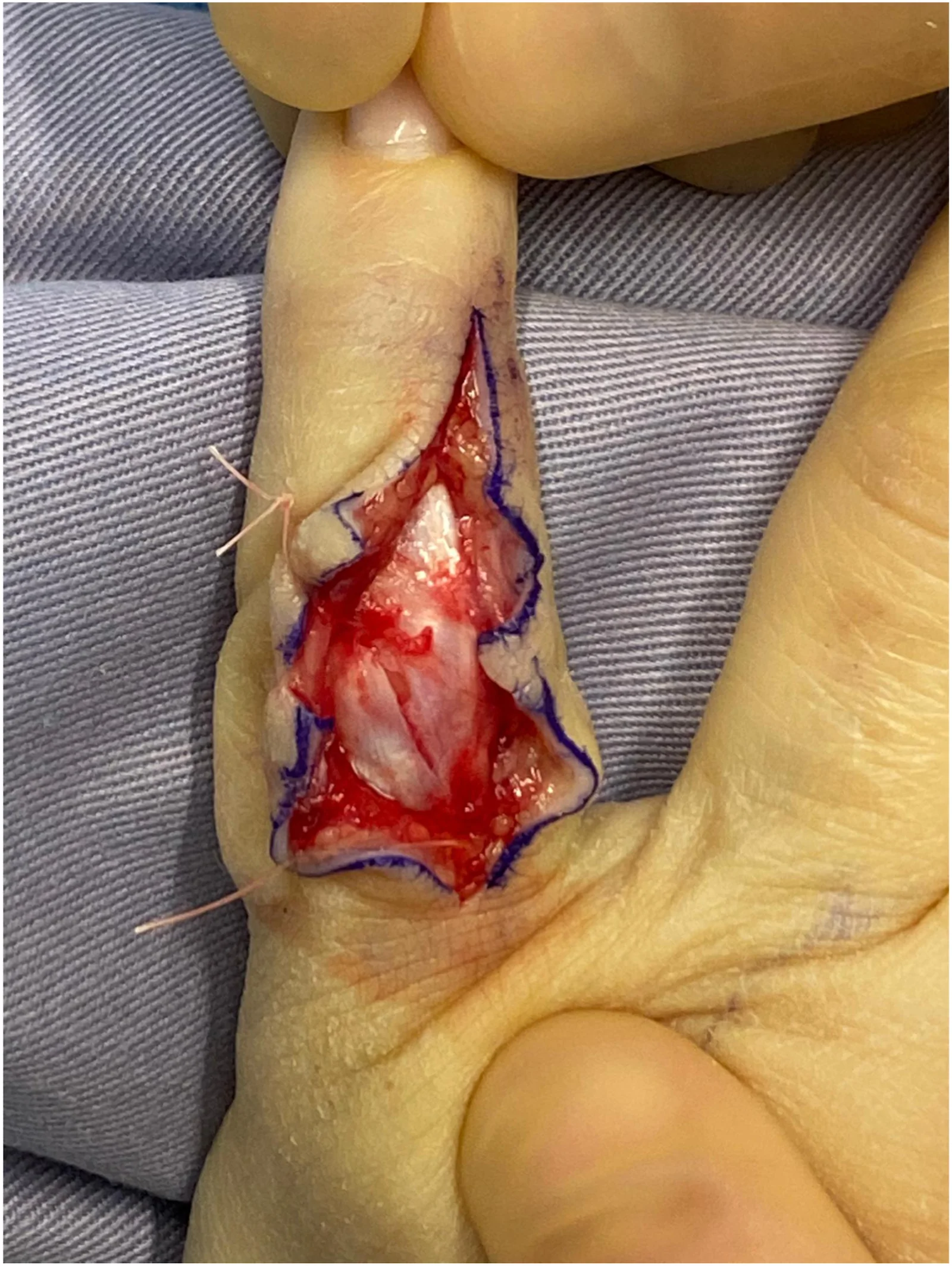

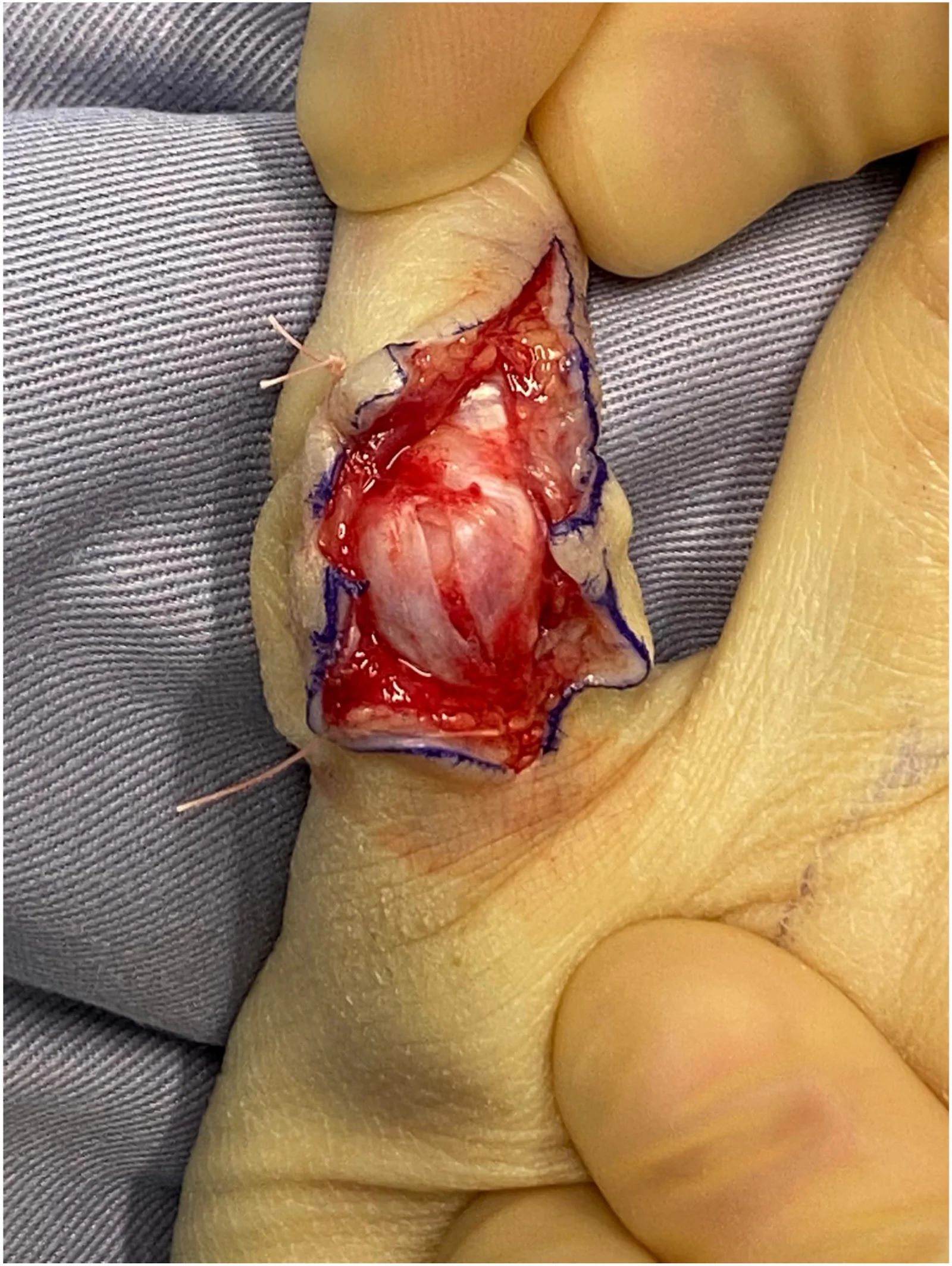

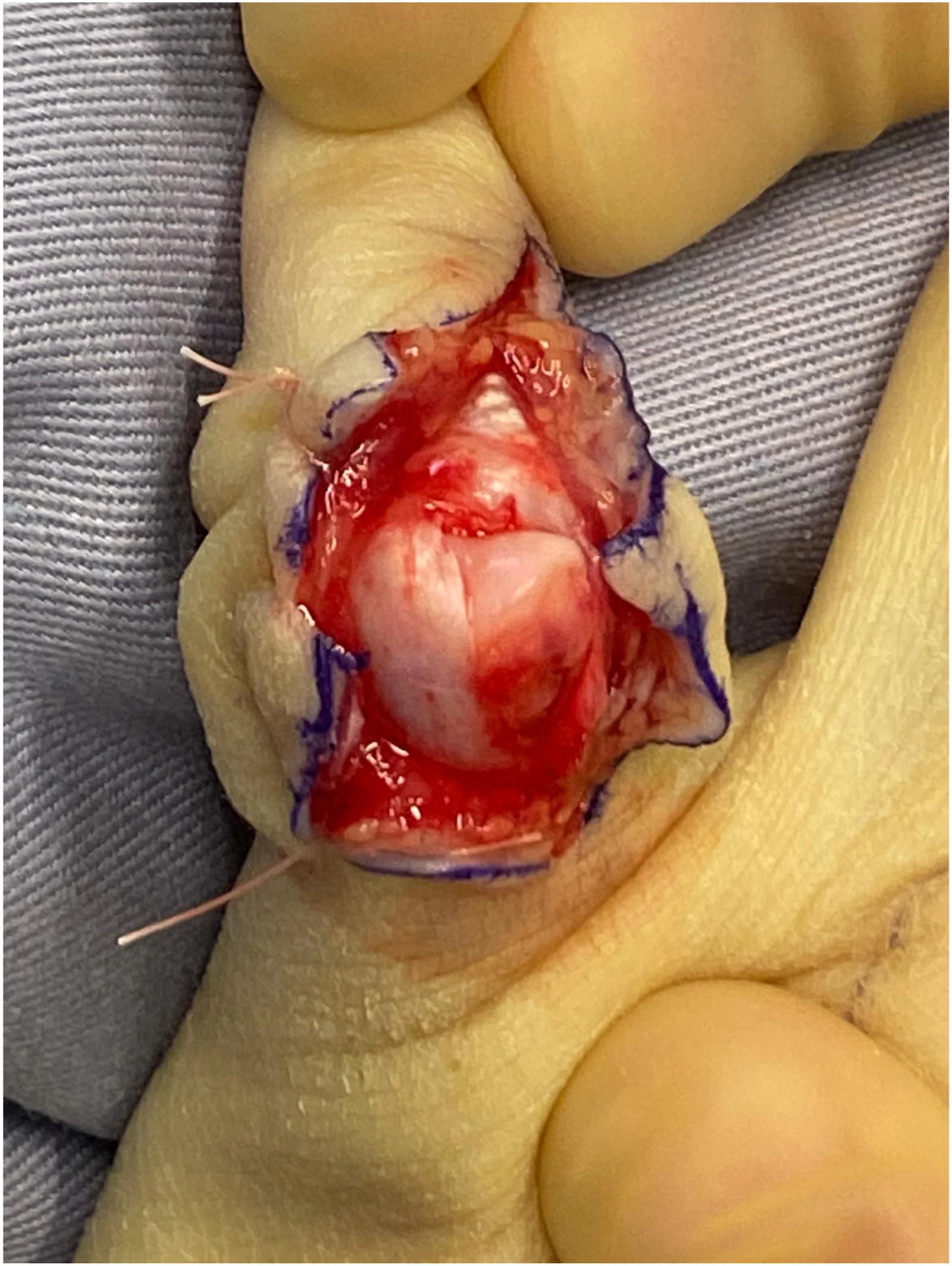
a) Longitudinal split of the radial lateral band in the clinical case, b) and c) Palmar, unreducible migration after flexion of the PIP joint.

Intraoperative exploration revealed a longitudinal tear of the extensor apparatus between the central slip and the radial lateral band at the level of the PIP joint. The central slip itself remained largely intact. The radial lateral band was found to be displaced palmarly beneath the level of the PIP joint axis, without interposition between the articular surfaces and without palmar subluxation of the joint. No intraoperative evidence of volar plate injury was identified. Repositioning of the lateral band to its anatomical dorsal position was only achievable using forceps. Passive flexion of the joint reproducibly caused recurrent palmar displacement of the radial lateral band, resulting in an immediate boutonniere-type deformity (Fig. 7 and Online Supplemental Video S6). A complete rupture of the radial collateral ligament was confirmed, explaining the associated lateral instability.

Supplementary video related to this article can be found at doi:10.1016/j.jham.2026.100490

The following are the supplementary data related to this article:

S6: In vivo demonstration of the specific pattern of lesion described in the case report, with palmar dislocation of the longitudinally damaged radial lateral band after PIP joint flexion.

No pseudo-Stener-like effect was observed.

**Fig. 8:**
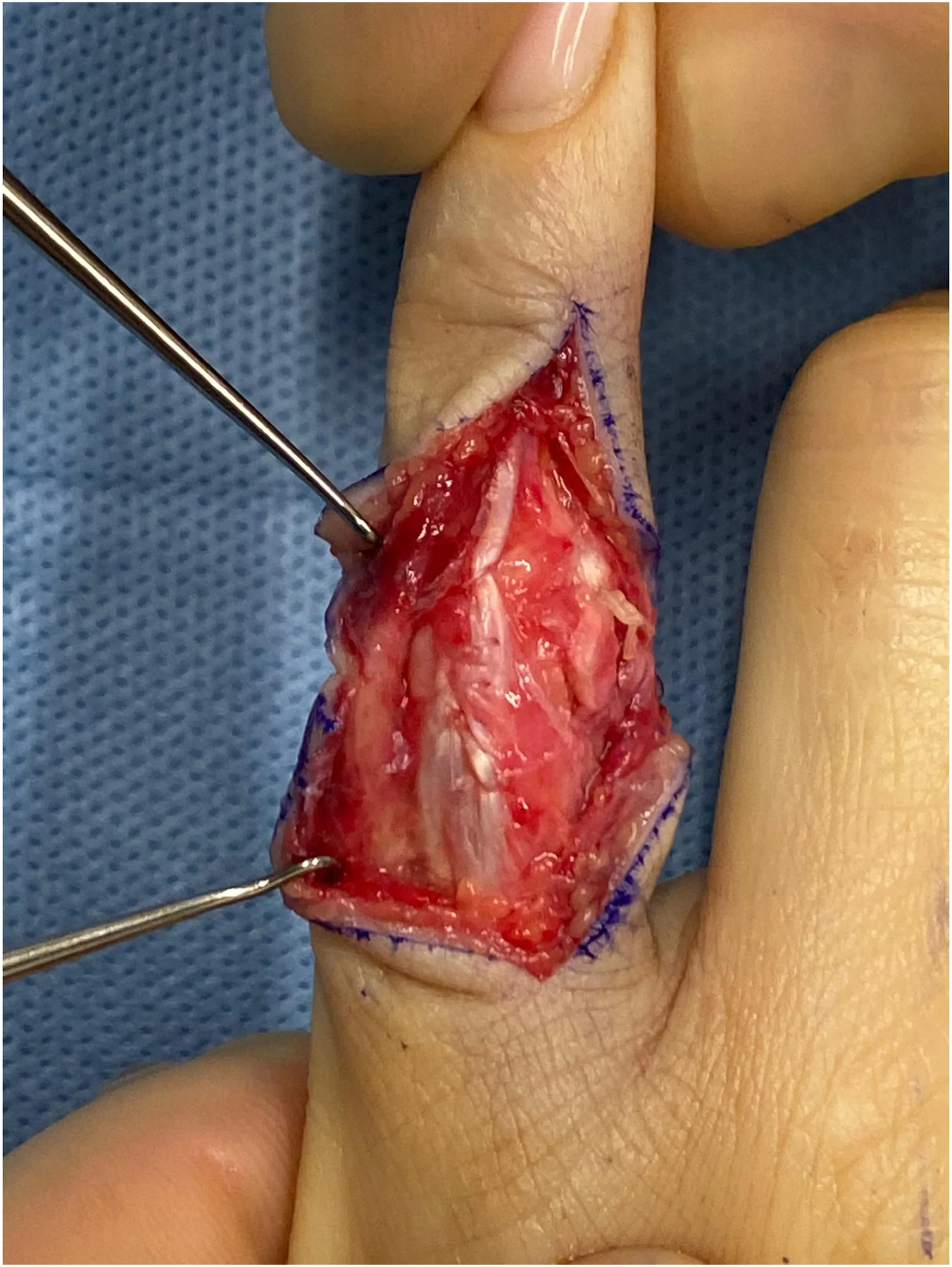
Extensor tendon repair consisting only of a longitudinal running suture, without reattachment of the central slip, and with refixation of the collateral ligament (the latter is not shown).

Surgical treatment consisted of direct repair of the longitudinal extensor tendon tear using a running interlocking suture technique with absorbable material, without the use of bone anchors (Fig. 8 and Online Supplemental Video S7). The radial collateral ligament was subsequently reattached, restoring lateral stability of the PIP joint.

Supplementary video related to this article can be found at doi:10.1016/j.jham.2026.100490

The following are the supplementary data related to this article:

S7: Passive finger movement in case presentation after longitudinal tendon repair.

During postoperative. Rehabilitation, the PIP joint was protected in near-extension for the first two weeks. From the third postoperative week we initiated graded active and active-assisted PIP and DIP motion within an RMF orthosis for an additional four to six weeks, in line with the relative motion concept.

**Fig. 9:**
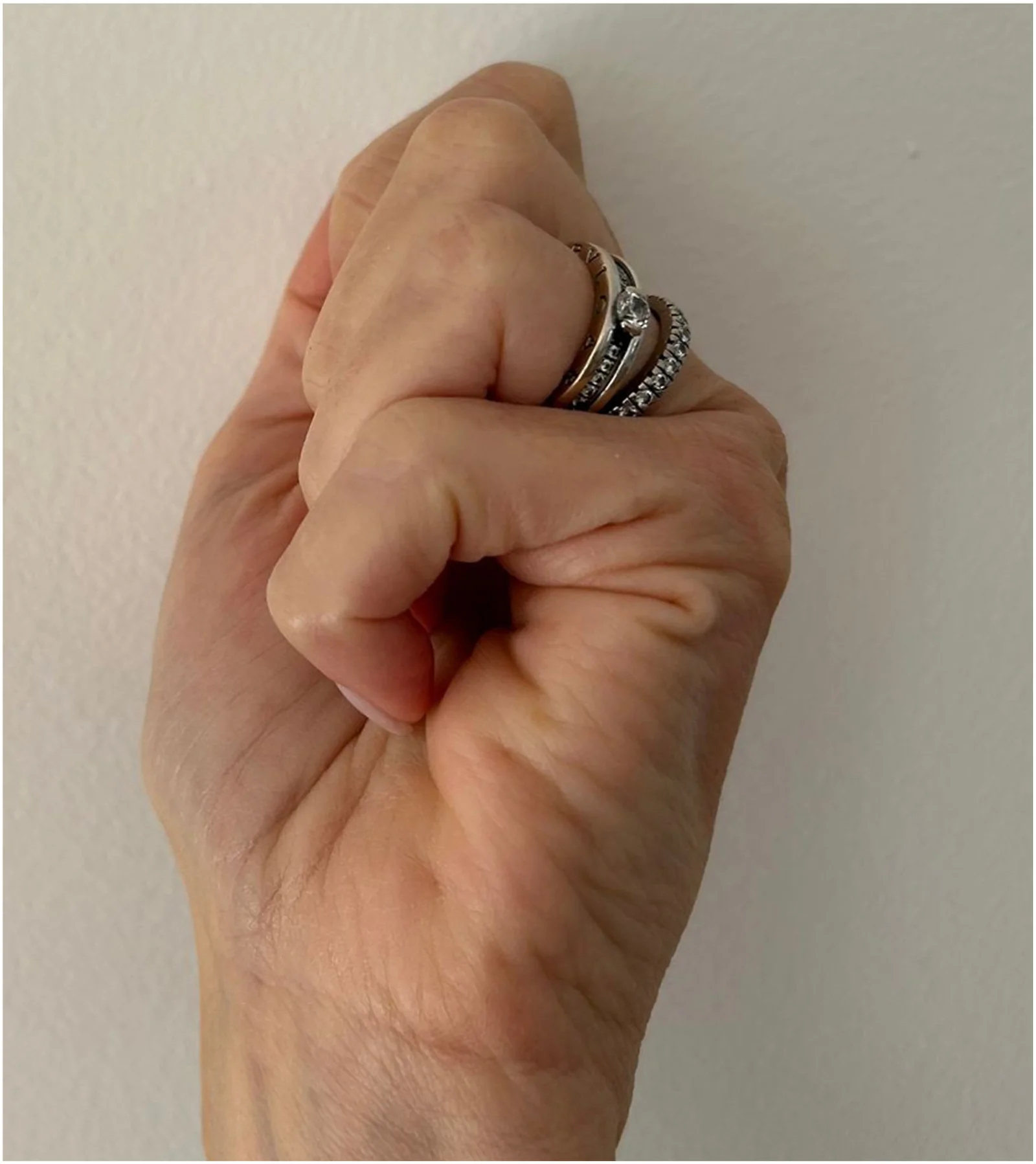
Finger flexion at 6 months, without boutonniere deformity and with full DIP joint flexion.

At follow up joint stability and extensor mechanism balance were restored, with resolution of the ulnar instability and correction of the boutonniere deformity. A mild residual swan-neck deformity was observed. However, this did not impair overall hand function (Fig. 9 and Online Supplemental Video S8). The residual deformity was therefore attributed to post-repair imbalance and healing-related stiffness within the extensor mechanism rather than to an unrecognized volar plate lesion.

Supplementary video related to this article can be found at doi:10.1016/j.jham.2026.100490

The following are the supplementary data related to this article:

S8: Finger function six months after repair. A slight hypercorrection is seen with swan-neck posture.

## 5 Discussion

### 5.1 What is known

Boutonniere deformity is classically described as flexion of the PIP joint with hyperextension of the DIP joint, secondary to dysfunction of the central slip of the extensor mechanism and subsequent palmar displacement of the lateral bands.^1^, ^2^, ^3^ Most current teaching places central slip detachment, with variable involvement of the triangular ligament and the spiral fibers of the intrinsic hood, at the center of the pathomechanics, with progressive palmar migration of the lateral bands occurring over time.^6^, ^7^, ^8^^,^^16^, ^17^, ^18^, ^19^ The time required from central slip detachment and lateral bands palmar migration is unknown.

In acute closed injuries, the terms “central slip detachment” and “acute boutonniere” are often used interchangeably, and the diagnosis traditionally relies on a combination of clinical signs and specific tests. The Elson test^5^ and its modifications are widely used to detect central slip incompetence. However, Houston et al. have shown that several hundred flexion–extension cycles may be required before a positive test reliably appears, which is rarely realistic in a freshly injured, painful finger.^20^ The pencil test, in which the patient is asked to actively extend the PIP joint against a pencil placed across the dorsal aspect of the proximal phalanges, is a simple way to assess residual active PIP extension and is used as an entry criterion for relative motion flexion (RMF) treatment of acute closed boutonniere.^21^^,^^22^ The Boyes test, in which the inability to flex the DIP with the PIP held in extension is interpreted as a tight lateral band system, helps to distinguish a true boutonniere deformity from a pseudoboutonniere deformity due to volar plate injury.^23^

In patients with full passive PIP extension and a positive pencil test, RMF orthoses have been shown to allow active hand use during healing of the central slip and yield favorable functional outcomes both in acute and in selected chronic cases, including those treated with preliminary serial casting to neutral followed by 12 weeks of RMF management.^21^^,^^22^ Conversely, an acute, irreducible deformity that cannot be corrected by closed means has been reported in the context of irreducible volar rotatory subluxation of the PIP joint, where one collateral ligament is disrupted, the ipsilateral lateral band is displaced palmar to the joint axis.^13^, ^14^, ^15^ These injuries have generally required surgical exploration.

Anatomically, the central slip has historically been described as inserting onto the dorsal tubercle of the middle phalanx. As pointed out by Kaplan, however, a substantial portion of the central slip blends with the dorsal capsule of the PIP joint rather than inserting discretely on bone.^24^ This continuity is still relatively under-emphasized in textbooks and clinical practice, where the central slip is often depicted as a discrete bony insertion.

### 5.2 What this study add

Within the limits of an exploratory cadaveric model and a single illustrative clinical case, the present work adds the following observations. First, in our specimens, isolated transverse detachment of the central slip and isolated longitudinal disruption of a single lateral band including the triangular aponeurosis did not produce an actively irreducible boutonniere deformity. The lateral band returned to its anatomical alignment during simulated active extensor and intrinsic activation in all specimens. This is consistent with previous cadaveric and clinical observations indicating that an isolated central slip injury rarely causes an immediate, fixed deformity.^6^, ^7^, ^8^, ^9^, ^10^, ^11^, ^12^

The addition of an ipsilateral collateral ligament lesion was, in our model, consistently associated with immediate palmar migration of the lateral band below the PIP joint axis and with an actively irreducible boutonniere deformity, regardless of central slip integrity. The displacement of a single lateral band was enough to produce the deformity. This pattern reproduced the configuration observed intraoperatively in the presented clinical case, in which the central slip was largely intact and the deformity was driven by longitudinal disruption of a single lateral band combined with rupture of the ipsilateral collateral ligament.

**Fig. 10:**
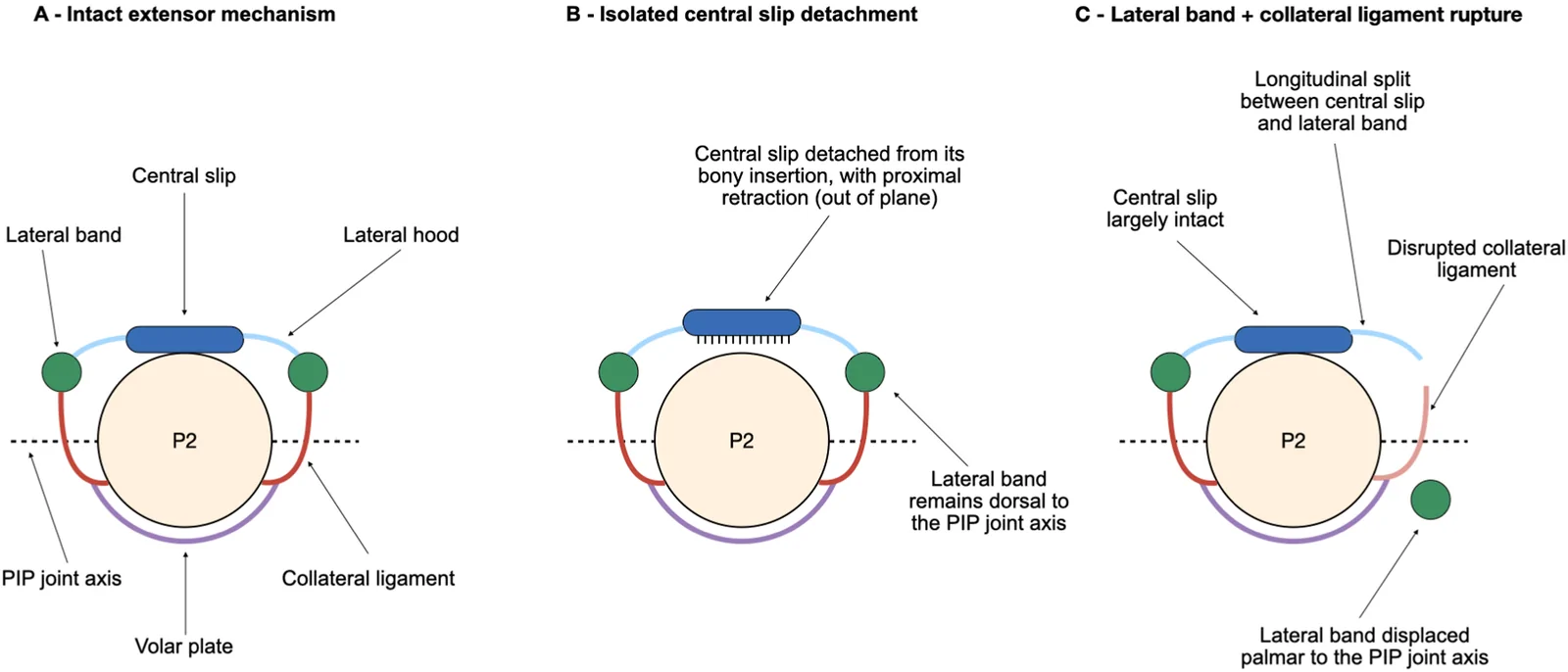
Schematic representation of the proposed mechanism of acute boutonniere deformity, drawn as an axial cross-section through the proximal interphalangeal (PIP) joint at the level of the head of the proximal phalanx. (A) Intact extensor mechanism: the lateral band (green) lies dorsal to the PIP joint axis, supported palmarly by the collateral ligament (red), with the central slip (blue) attached to the bone and connected to each lateral band by the lateral hood (light blue); the volar plate (purple) closes the joint palmarly. (B) Isolated central slip detachment: the central slip is detached from its bony insertion and tends to retract proximally, out of the section plane; the lateral bands remain dorsal to the PIP joint axis. (C) Combined longitudinal disruption of one lateral band and rupture of the ipsilateral collateral ligament: the central slip itself remains largely intact, but the lateral hood between the central slip and the lateral band is split; the lateral band, no longer supported palmarly by the collateral ligament, migrates palmar to the PIP joint axis, producing an immediate, actively irreducible boutonniere deformity. P2: middle phalanx (sectioned). Dashed line: PIP joint axis.

Third, the loss of the collateral ligament appeared to have a relevant biomechanical role beyond joint stability. The bulk of the collateral ligament constitutes the principal palmar supporting structure of the lateral band, as illustrated anatomically by van Zwieten et al.^25^ and represented in Fig. 10. Its disruption removed this palmar buttress and, in our specimens, allowed the lateral band to migrate beneath the joint axis. This may extend the concept of irreducible volar rotatory subluxation, previously described primarily in terms of joint kinematics and radiographic alignment,^13^, ^14^, ^15^ by emphasizing the parallel consequence on the extensor mechanism rather than the joint.

Finally, in our specimens we consistently observed marked physiological thinning of the central slip just proximal to its attachment, where it became continuous with the dorsal capsule of the PIP joint (Online Supplemental Video S5). This supports the concept, originally raised by Kaplan,^24^ that the central slip functions in part as a capsular structure connecting the lateral bands rather than as a purely tendinous bony insertion. The intraoperative finding that direct repair of the longitudinal split of the extensor apparatus, without reattachment of the central slip to bone, restored extensor balance in our patient is consistent with this anatomical view.

### 5.3 What remains uncertain

Several issues are not resolved by the present work. The exact in vivo timing of palmar displacement of the lateral band after isolated central slip rupture remains undefined in relation to the classical interpretation of boutonniere deformity. The present model does not directly address this question, as it investigates a different type of mechanism for boutonniere.

The relationship between the “irreducible volar rotatory subluxation” described on radiographs, and the configuration we observed (in the absence of bony subluxation or soft tissue interposition between the articular surfaces) warrants further clarification, ideally through prospective imaging studies.

Indeed, the concept of volar rotatory subluxation itself remains incompletely characterized in the literature, with limited objective description of the actual displacement occurring at the PIP joint. It is conceivable that, in at least some reports, the marked flexion posture of an acute boutonniere deformity was interpreted as a volar subluxation despite the absence of true translational displacement of the articular surfaces. Whether these represent distinct pathological entities or different descriptions of the same deformity remains uncertain.

In our clinical case and throughout our study, we did not observe palmar subluxation of the joint, even in the experimental group in which the central slip was detached from the middle phalanx. If a subset of acute boutonniere deformities results from the configuration observed in our clinical case and in Group 2, it is difficult to understand why the PIP joint would dislocate palmarly, as the dorsal stabilizers remain largely intact.

Conversely, it is also plausible that, for an active irreducible boutonniere deformity to occur in the manner we describe, the causative trauma is most likely directed volarly with a rotational component that disrupts the collateral ligaments.

The proportion of acute closed boutonniere presentations in everyday practice that correspond to a combined lateral band/collateral ligament injury, rather than to an isolated central slip lesion, is currently unknown. Whether the injury pattern described here merits explicit recognition as a distinct subset of acute boutonniere deformity will ultimately depend on additional clinical and imaging data.

### 5.4 Clinical implications

Our observations, taken together with the existing literature, suggest a pragmatic stepwise approach to the patient presenting acutely with a post traumatic flexed PIP joint and impaired active extension. The first step is to assess passive PIP extension and lateral PIP joint stability. When passive extension is full and the joint is laterally stable, the pencil test as described by Merritt and Jarrell is used: a pencil, tongue blade or even digital pressure is placed across the dorsum of the proximal phalanx of the injured and adjacent digits to keep the injured MP joint 15°–20° more flexed than the neighbors. If full active PIP extension is now possible, the deformity is considered correctable, and a relative motion flexion (RMF) orthosis with active hand use is appropriate for approximately six weeks.^21^

The Elson test^5^ and its modifications^20^ can be used as adjuncts to detect central slip incompetence, with the caveat that several hundred flexion-extension cycles may be required for a freshly ruptured central slip to give a reliable Elson sign. The Boyes test, performed by comparing passive DIP flexion with the PIP fully extended versus fully flexed, becomes most informative in subacute and chronic presentations, when fixed proximal retraction or palmar displacement of the lateral bands has had time to develop. It also helps to differentiate a true boutonniere deformity from a pseudoboutonniere deformity related to a volar plate or pulley injury.^21^^,^^23^

When the PIP joint shows clinically relevant lateral instability, or when the lateral band cannot be brought back above the joint axis on passive manipulation (so that the deformity recurs on subsequent flexion despite a passively extensible joint), the present work supports a low threshold for additional imaging. Ultrasound, in our case, allowed early identification of the rupture of the radial collateral ligament and of displaced ligamentous and tendinous structures, and was instrumental in deciding for surgical exploration. We therefore consider focused ultrasound a useful adjunct in this subgroup, with attention to (i) the integrity of the collateral ligaments, (ii) the position of the lateral bands relative to the PIP joint axis, and (iii) the continuity of the central slip and triangular ligament. MRI may be considered when ultrasound is inconclusive or in chronic presentations.

Patients in whom imaging or examination identifies combined longitudinal lateral band disruption and collateral ligament rupture should, in our opinion, be considered for early surgical exploration, since this configuration was actively irreducible in our cadaveric specimens and required surgical correction in our clinical case. Within the limits of a single observation, simple longitudinal repair of the extensor apparatus combined with refixation of the collateral ligament was sufficient to restore extensor balance, without the need for reattachment of the central slip to bone. Alternatively, provided that only one lateral band is palmarly migrated, after the collateral ligament has been reattached, then a tenotomy of the affected lateral band could be performed, instead of a repair.

Where feasible, the wide-awake local anesthesia no tourniquet (WALANT) technique can be considered: it allows intraoperative assessment of intrinsic balance with the patient awake and actively cooperating, which is particularly useful when restoring the delicate extrinsic-intrinsic balance is the principal aim. We acknowledge that the optimal rehabilitation protocol after this specific injury pattern remains to be defined.

**Fig. 11:**
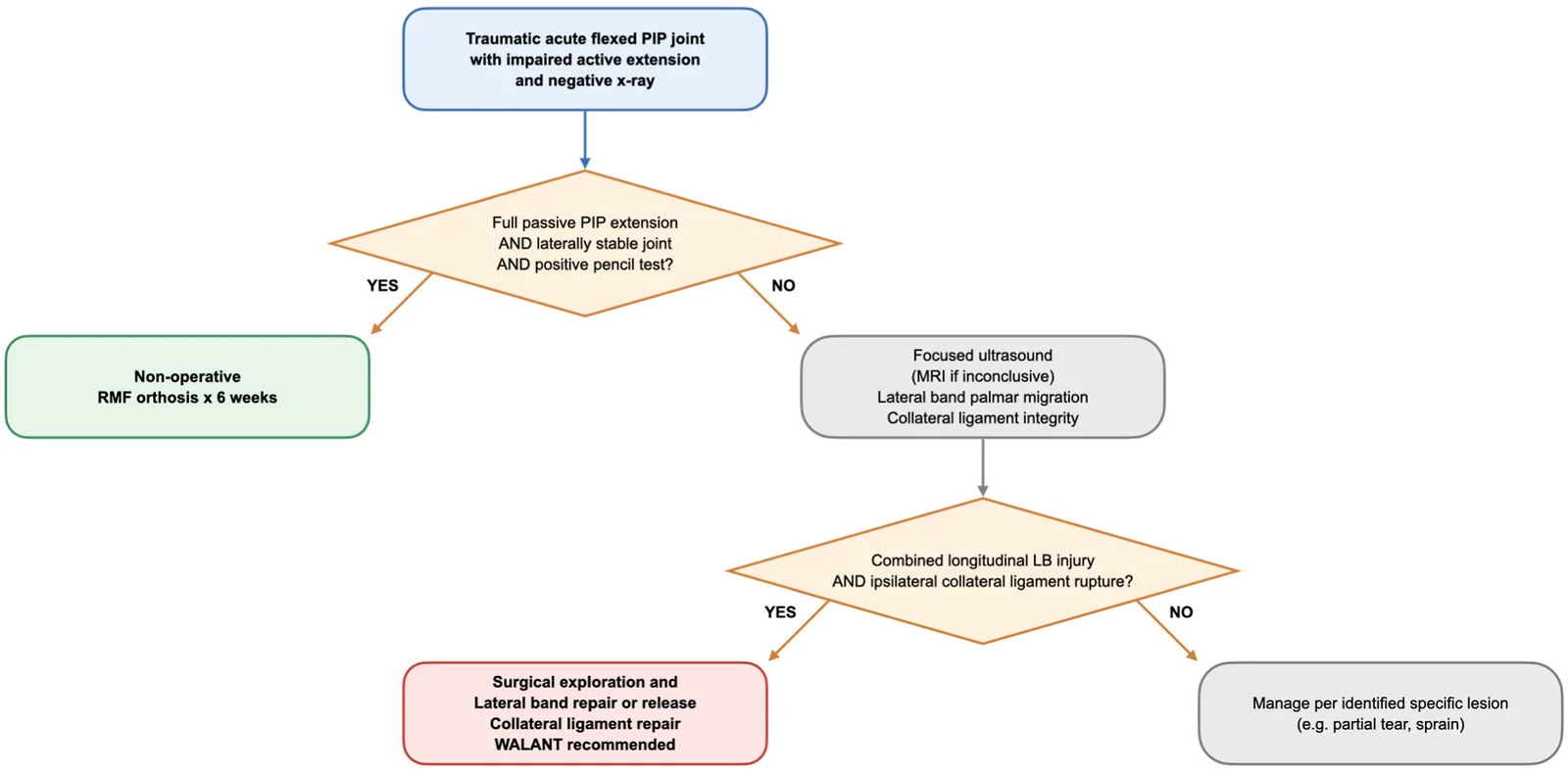
Proposed diagnostic and management framework for the patient presenting with a traumatic acute boutonniere deformity.

Based on these considerations we propose, on a hypothesis-generating basis, a simple practical framework for the acute presentation, summarize in Fig. 11 (diagnostic and management flow chart), to be validated in future prospective work. The flow chart applies to a traumatic, acutely flexed PIP joint with impaired active extension and a negative radiograph (no fracture, no joint subluxation), and distinguishes two pathways: (1) a finger with full passive PIP extension, lateral stability and a positive pencil test, manageable non-operatively with an RMF orthosis for approximately six weeks, and (2) a finger with lateral instability or with a lateral band that cannot be repositioned dorsally above the joint axis on passive maneuvers, in which focused ultrasound (or MRI when inconclusive) is used to look for combined longitudinal lateral band injury and ipsilateral collateral ligament rupture, with the option of surgical exploration with collateral ligament repair plus either repair or selective release of the affected lateral band, performed under WALANT where feasible.

### 5.5 Limitations

This study has several important limitations. The cadaveric sample is small (16 fingers from four hands) and was studied in vitro. Cadaveric tissue does not fully reproduce the active behavior of intrinsic and extrinsic muscles, the contribution of skin and subcutaneous tissues, or proprioceptive feedback.

Our injury simulations are experimental and may not exactly match the spectrum of lesion patterns encountered in vivo.

The clinical component is a single case report. While it permitted direct intraoperative correlation with the cadaveric findings, it cannot, by itself, support generalized conclusions.

Finally, our observations on the capsular character of the central slip are qualitative and require confirmation by dedicated histological and imagine studies. The present findings should therefore be regarded as exploratory and hypothesis-generating, and their clinical relevance must be confirmed in prospective in vivo series.

## Contributorship

All authors participated in the study design and manuscript drafting, all authors reviewed and edited the manuscript and approved the final version of the manuscript.

## Ethical approval

Ethical approval for this study was obtained from the Ethikkommission Nordwest-und Zentralschweiz (2024-02327).

## Informed consent

Written informed consent was obtained from the donors and the patient for their anonymized information to be published in this article.

## Funding

The authors received no financial support for the research, authorship, and/or publication of this article.

We would like to disclose that the GPT-4 model (ChatGPT, OpenAI) was used exclusively for language editing purposes to improve the clarity and fluency of the English text. No content-related assistance was provided.

## Declaration of competing interest

The authors declare that they have no known competing financial interests or personal relationships that could have appeared to influence the work reported in this paper.
